# Supplementary material for: Neuronal tuning and population representations of shape and category in human visual cortex
Source: Nat Commun. 2024 May 30;15:4608. doi: 10.1038/s41467-024-49078-3 (PMC11139926; doi:10.1038/s41467-024-49078-3)
Supplement: Supplementary file 1 — Supplementary Information [file 41467_2024_49078_MOESM1_ESM.pdf]

Single channel analysis – High-Gamma (HG) results: Out of the 332 visually responsive HG sites, 79 sites were significantly selective for the shape type dimension alone, whereas 19 were category – selective, and 100 had interactions between shape type and category ($chi2 = 66.8$, $p < 0.00001$). Note that a small number of channels ($N = 1$ and $N = 2$ in array 2 and 3, respectively) did not show any main effect or interaction effect in the 2-way ANOVA, but were nevertheless stimulus-selective based on a 1-way ANOVA on the net responses to all 54 stimuli.

Event Related Potential (ERP) analysis: To conduct the same 2-way ANOVA analysis at the single-channel level as performed for MUA and HG, we identified the time point corresponding to the initial peak of the ERP after the baseline for each visually responsive site. Subsequently, we calculated the median of all these time points and selected a time window of 25 milliseconds before and 25 milliseconds after that median point, as indicated by the green line in Figure S7. We then averaged the activity within this window for each channel and used the average to perform the single-channel analysis.

Decoding – HG results: We obtained similar results when decoding shape type and category using the high-gamma responses (Fig S8A); however, in comparison to the MUA decoding a few differences were found. Array 2 and 3 exhibited reliable category decoding over a time window of several hundreds of ms (which was shorter than for the MUA decoding), while category decoding on array 1 barely reached significance (and was sustained for the MUA). Array 4 also barely reached statistical significance for both factors due to the low signal-to-noise ratio of the HG on this array.

Figure S1: Utah array locations

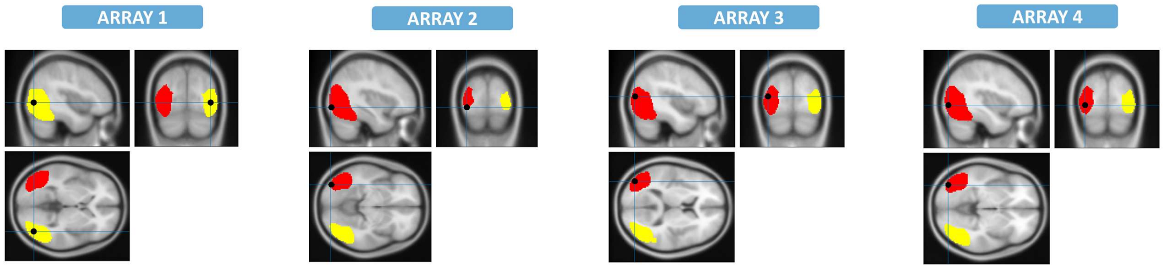

Figure S1: Left (yellow) and right (red) LOC parcels defined using the Group-Constrained Subject-Specific (GSS) method (Julian et al., 2012). Black circles indicate the location of each array.

Figure S2: Single Unit Activity – Example channels

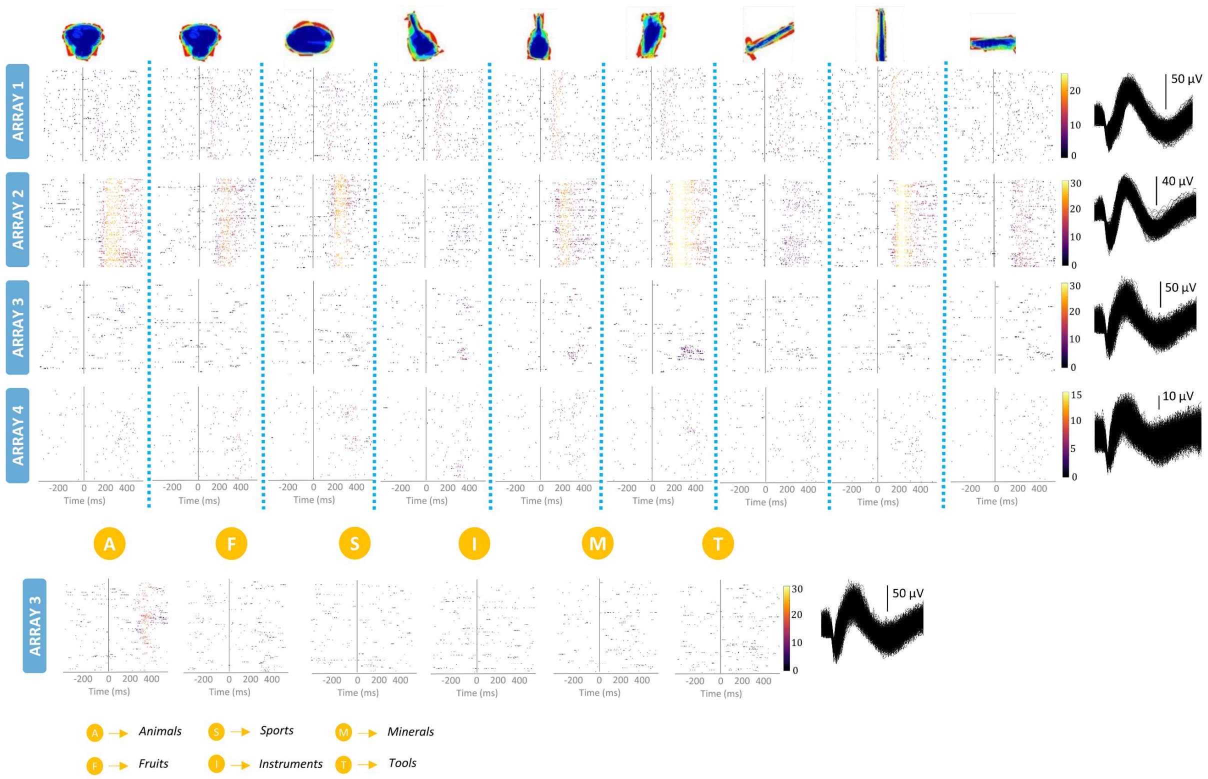

Figure S2: Raster plots and example waveforms of Single-Unit activity from one example channel per array. Rows represent trials, and columns depict different shapes (arrays 1, 2, 3, 4) or categories (array 3).

Figure S3: Selectivity

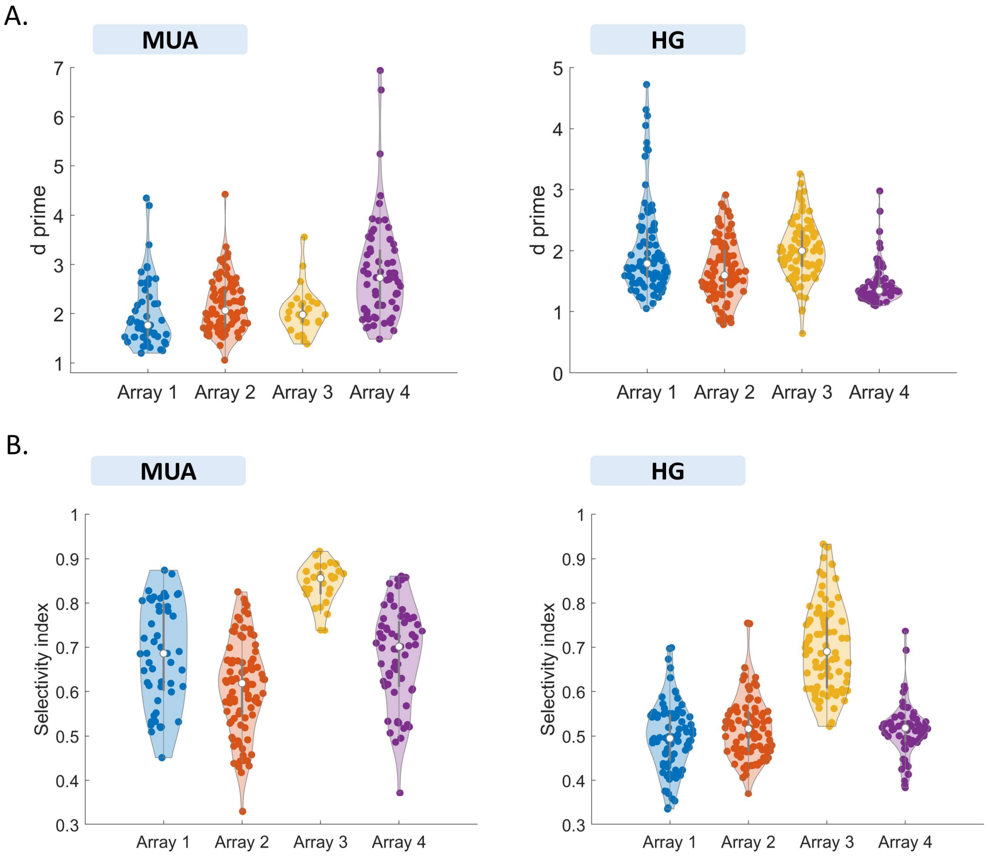

Figure S3: Violin plots depicting the distribution of d primes (A) for visually responsive channels in each array (left column MUA, right column HG) and Selectivity Indices (B).

Figure S4: Receptive Fields and Latency

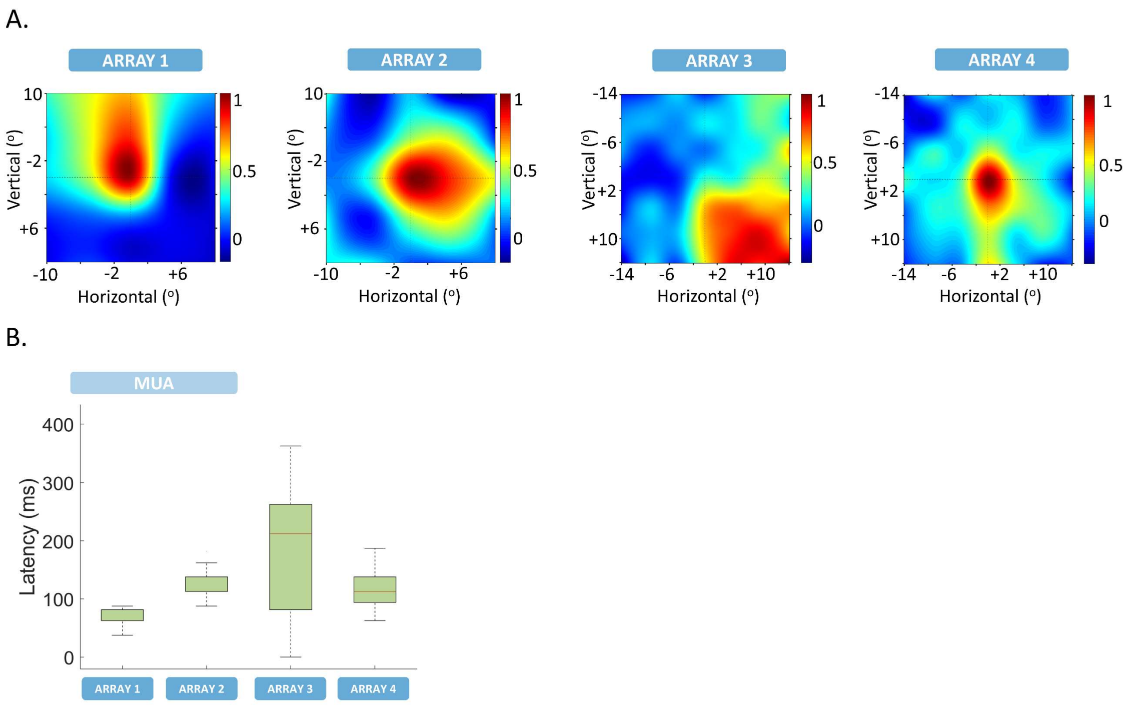

Figure S4: A) Average receptive fields across channels for each array. To map the receptive field (RF) of arrays 1,2, and 4, we showed a 4 deg flickering checkerboard (20 Hz) at 25 positions on the screen, covering a 10 x 10 degree area for arrays 1 and 2 and a 14 x 14 degree area of the visual field for array 4. In the case of array 3, instead of the checkerboard, we presented a 1 deg image of a body at 25 different positions, covering a 14 x 14 degree area of the visual field. **B)** Boxplots depicting the MUA latencies across channels per array. The top and bottom edges indicate the 75th and the 25th percentile respectively. The central line indicates the median and the whiskers extend to the most extreme data points (5th -95th percentile).

Figure S5: Responses to faces and taxonomical classes

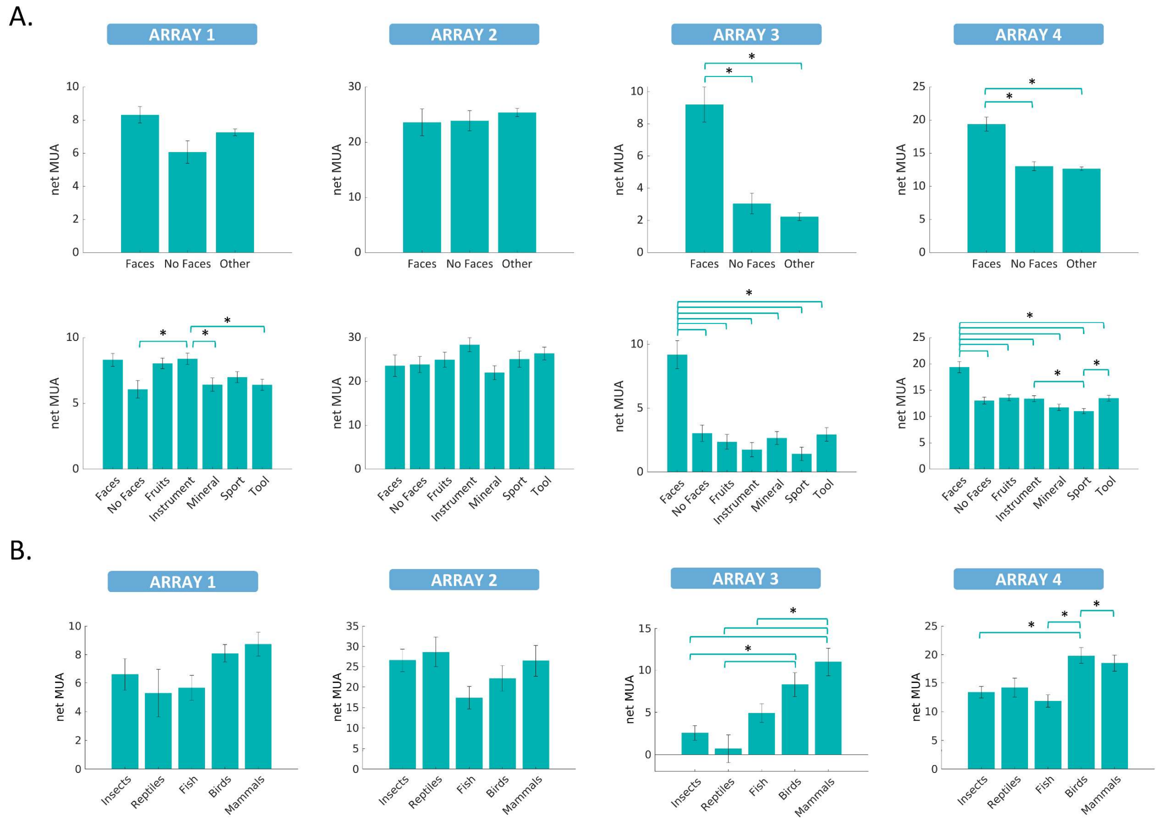

Figure S5: A) Average MUA activity across channels for each array—animals with faces, animals without faces, and other images (upper panel), and animals with faces, animals without faces, and individual other categories (lower panel). **B)** Average MUA activity across channels for each array for different taxonomical classes of animals (insects, reptiles, fish, birds, mammals). Asterisks (*) indicate significant differences following a one – way ANOVA, and error bars represent standard errors across channels.

Figure S6: Eta² values

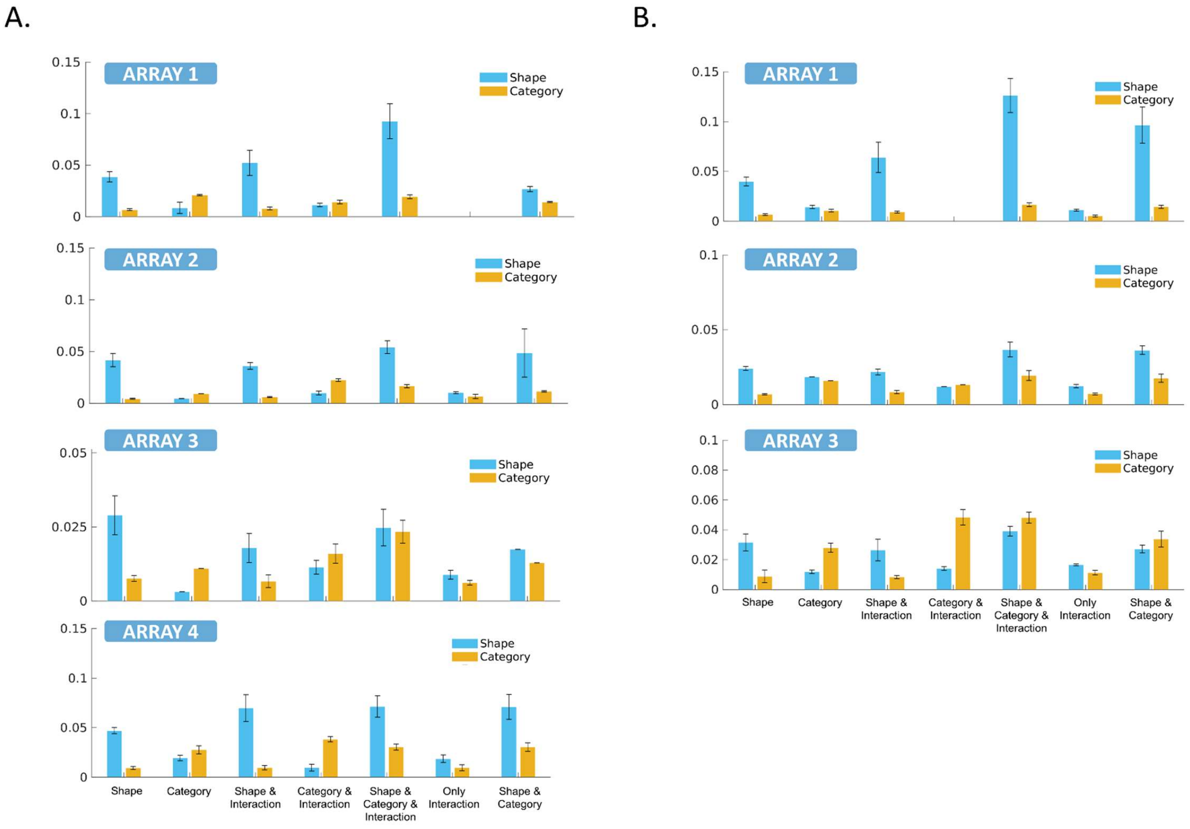

Figure S6: A) Average eta² values of the shape type (blue bars) and category (orange bars) dimension for all MUA sites with significant effects. The height of each bar indicates the average value across sites and the error bar represents the standard error. For arrays 1, 2, and 4, the eta² values for shape are consistently higher than those for category across all sites. In array 3, however, the difference is less pronounced for sites without a significant main effect of shape. **B)** eta² values for all high - gamma sites with arrays 1, 2, and 3 showing similar results to those observed at the MUA level. Array 4 is excluded due to a lack of channels with significant effects.

Figure S7: LFP – Event Related Potential

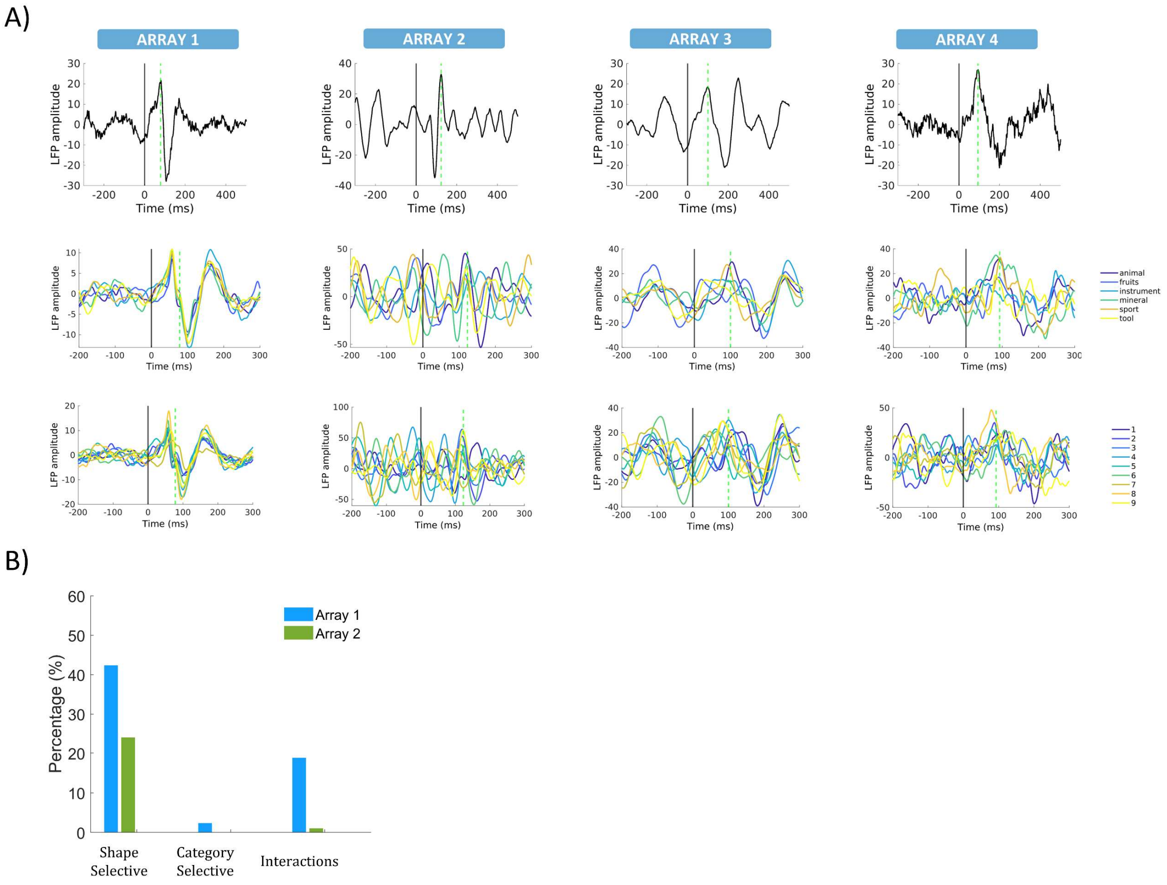

Figure S7: A) Average ERP across channels and stimuli (first row) and average ERP across channels plotted separately for each category (second row). Average ERP across channels plotted separately for each shape type (third row). The green line represents the median time point corresponding to the initial peak of the ERP after baseline. The selected time window for single-channel analysis spans 25 milliseconds before and after this median point. **B)** Summary of the results of the 2-way ANOVA (blue: Array 1, green: Array 2). The columns show the percentage of visually responsive channels with a significant effect of shape type, category, and interactions, respectively. Array 3 and 4 did not show any selectivity in the ERP.

Figure S8: Ranking, MUA – HG – ERP

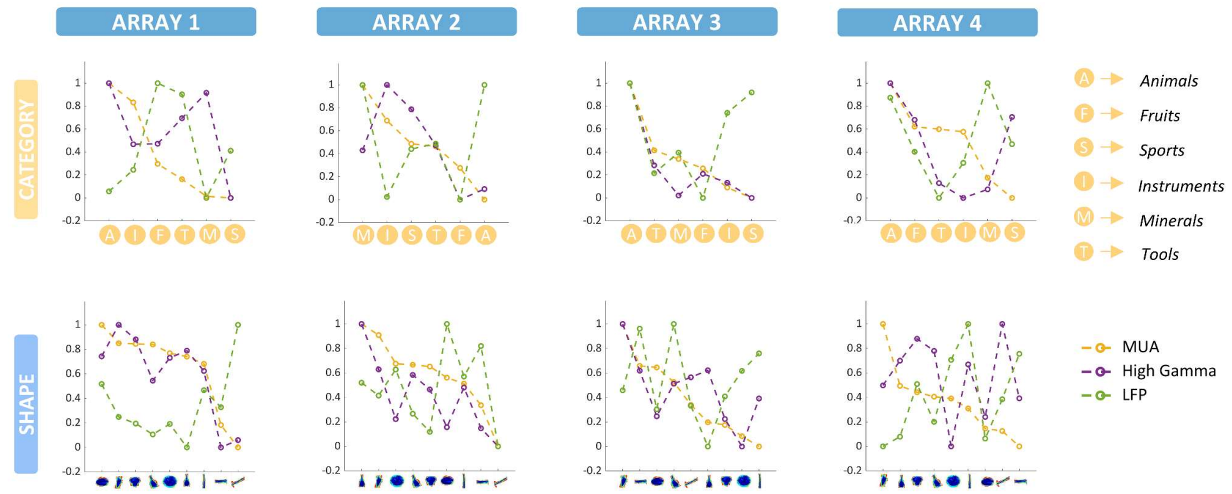

Figure S8: Ranking plots. The average normalized MUA (yellow dashed line) responses across channels were sorted in descending order separately for category (upper panel) and shape type (lower panel). The average High Gamma (HG) (purple dashed line) and Event-Related Potential (ERP) amplitudes (green dashed line) across channels were then plotted following that order.

Figure S9: Scatter plots – Correlation between neural and behavioral dissimilarity matrices

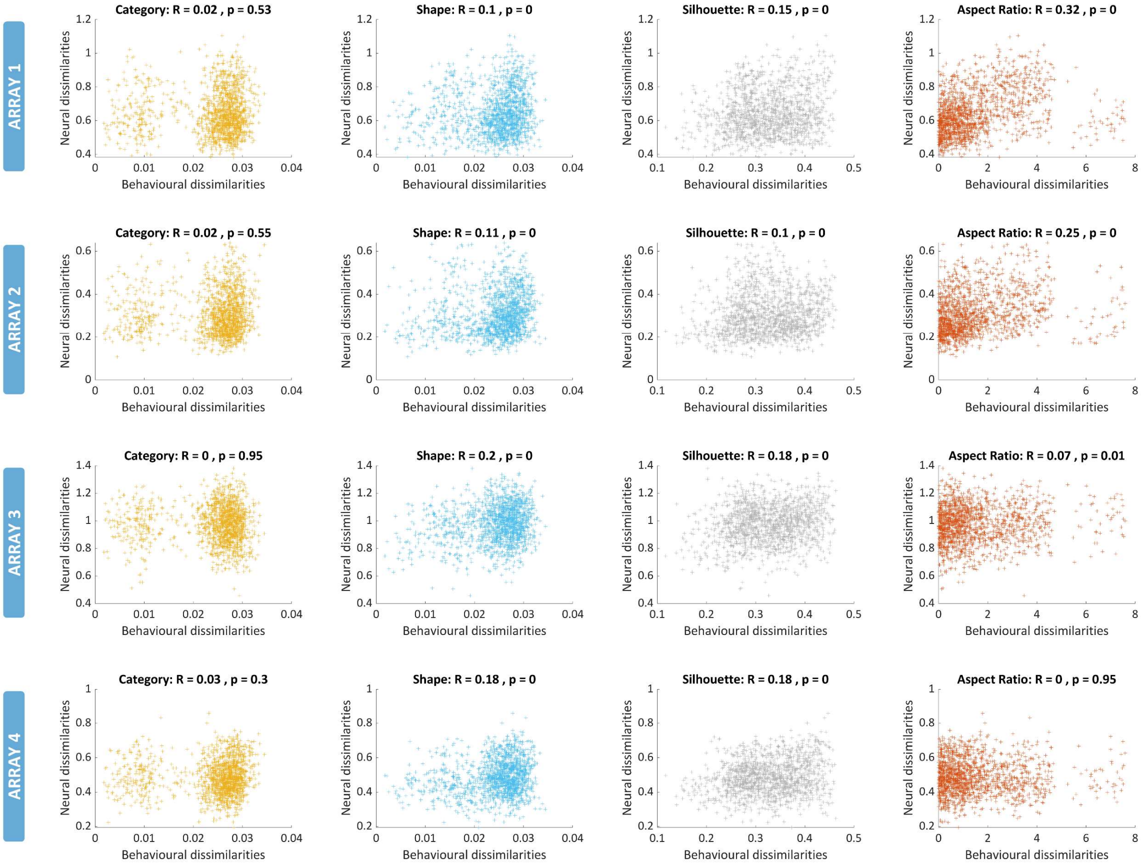

Figure S9: Scatter plots between neural dissimilarity matrices and behavioral (category and shape), silhouette, and aspect ratio dissimilarity matrices per array (individual rows) for each stimulus pair. The title in each plot provides the Pearson correlation and p-value.

Figure S10: Scatter plots – Correlation between aspect – ratio dissimilarity matrix and behavioral, and silhouette dissimilarity matrices

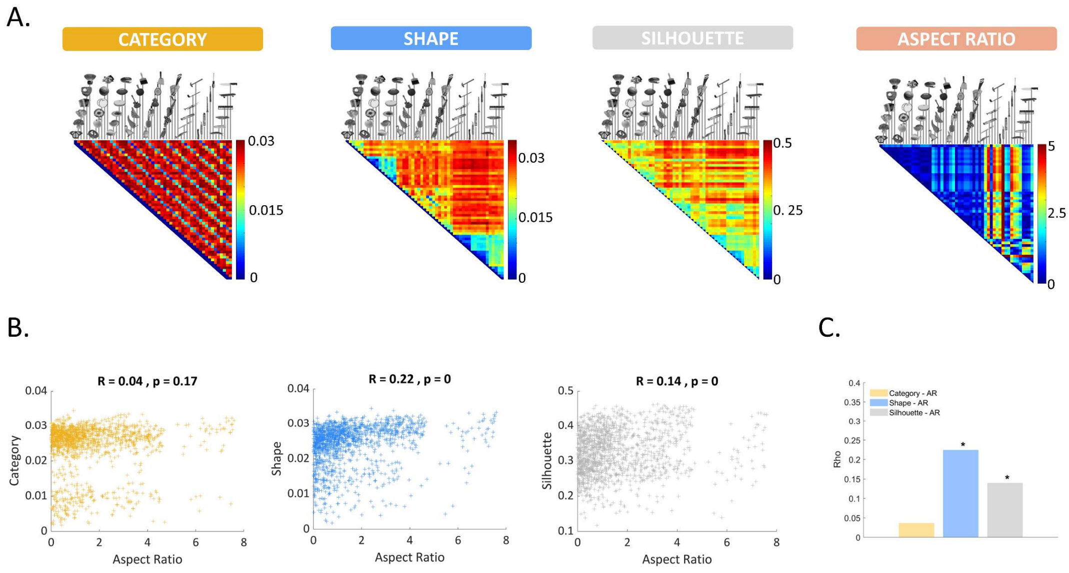

Figure S10: A) Color plots of the shape and category behavioral Representational Dissimilarity Matrices (RDMs), the silhouette RDM, and the aspect ratio RDM. **B)** Scatter plots indicating the correlation between the aspect-ratio RDM and the category (orange), shape (blue), and silhouette (grey) RDMs. The title in each plot provides the Pearson correlation and p-value. **C)** Barplots of the Pearson correlation between the aspect-ratio RDM and the shape, category, and silhouette RDMs. The height of each bar indicates the correlation, and asterisks (*) indicate significance.

Figure S11: Dissimilarity analysis for the high – gamma responses

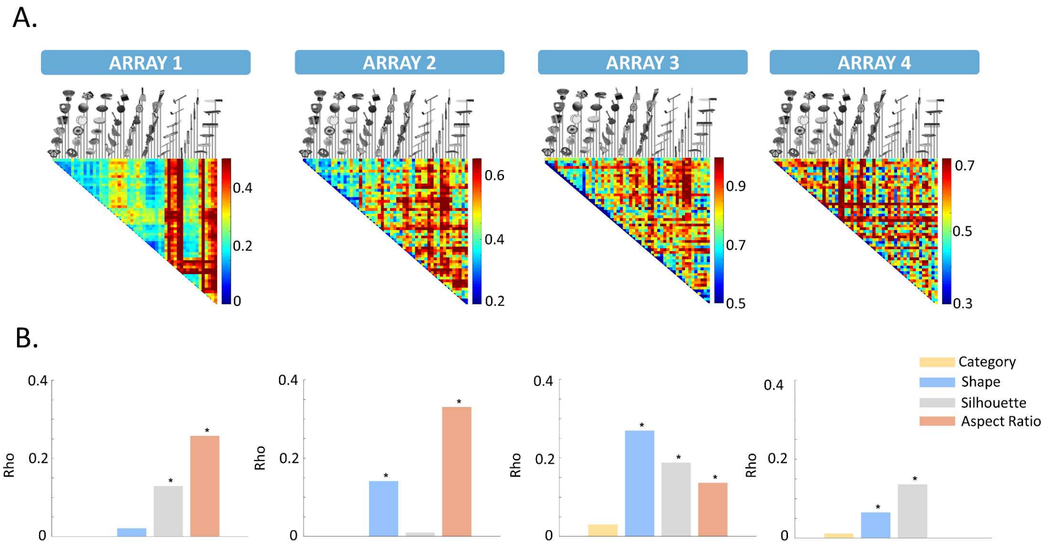

Figure S11: A) Neural RDMs for all arrays based on the high - gamma responses. **B)** Results of RSA for category – RDM (orange), shape – RDM (blue), silhouette – RDM (grey), and aspect – ratio RDM (red). The asterisks (*) indicate the significance of the correlation.

Figure S12: Multidimensional scaling statistics

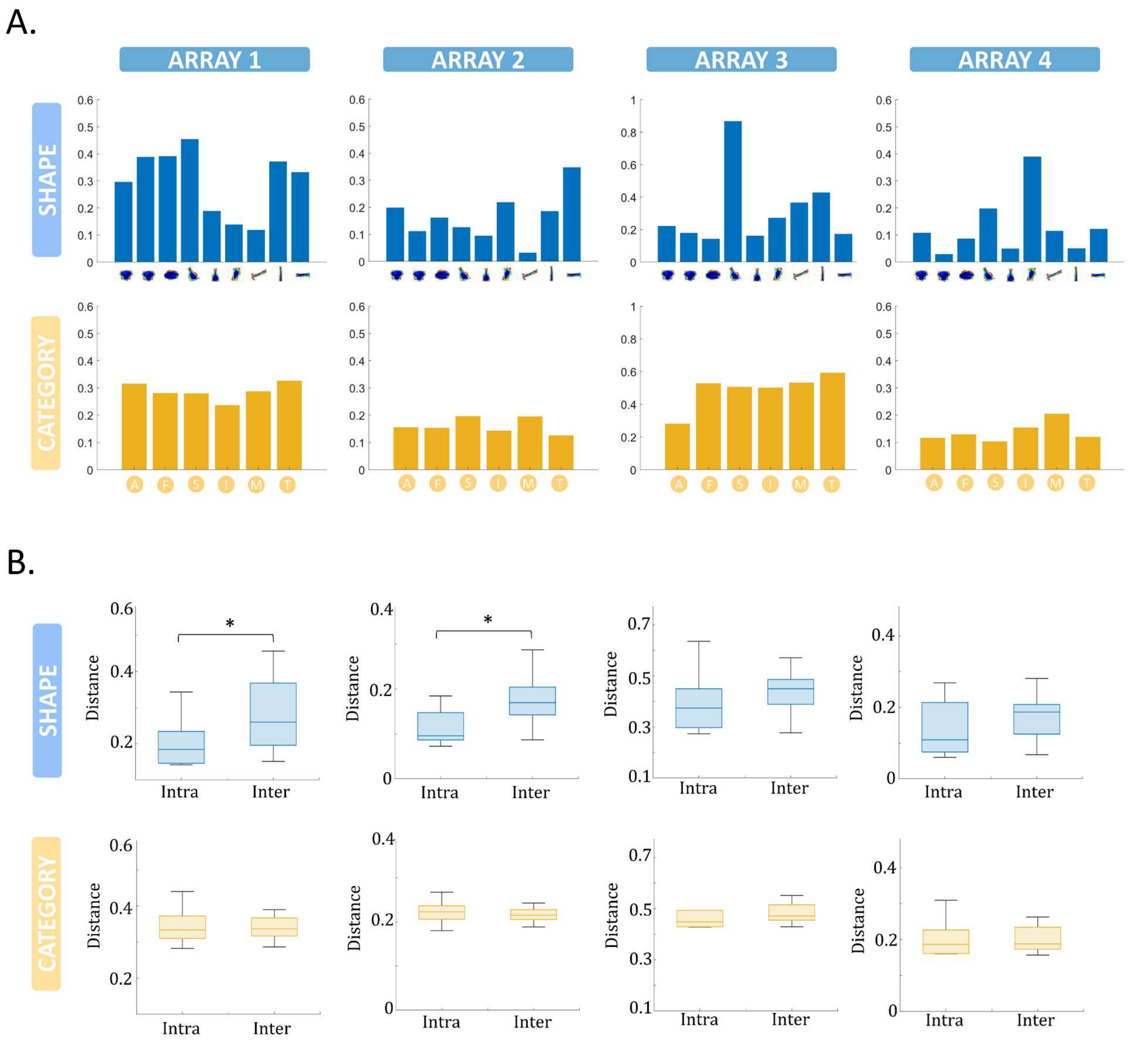

Figure S12: A) Barplots indicating the Euclidean distances between stimuli belonging to the same shape type (blue barplots) and between stimuli belonging to the same category group (orange barplots) within the 2-dimensional MDS space. **B)** Boxplots illustrating the Euclidean distances within (intra-cluster) and between (inter-cluster) conditions for each array, separately for the shape (blue boxplots) and category (orange boxplots) dimensions. Asterisks (*) indicate significant differences between inter- and intra-cluster distances following a one – way ANOVA.

Figure S13: Hierarchical cluster analysis

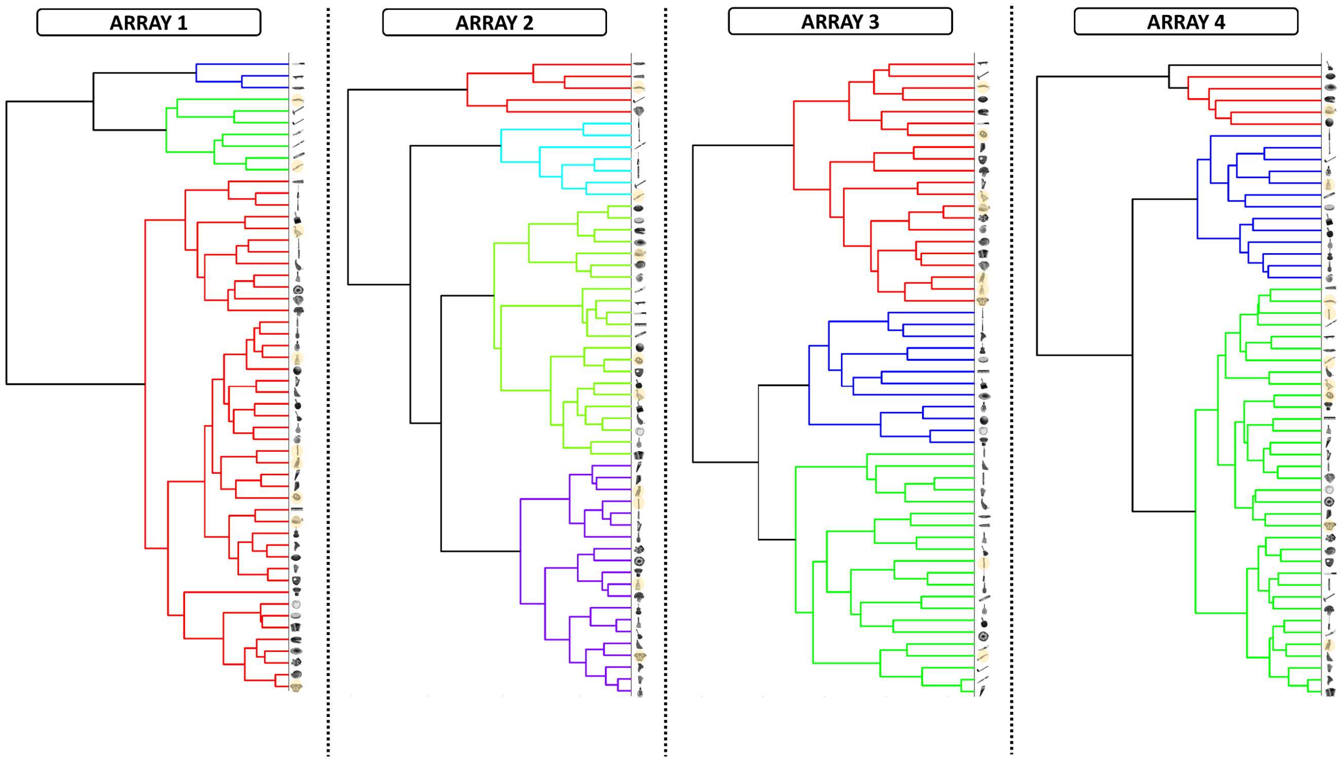

Figure S13: The hierarchical plot shows the structure of the neural dissimilarity matrix of each array. Results reveal clustering according to shape type for arrays 1,2, and 4, but not for array 3. Array 3 shows more clustering according to category, specifically for the "Animal" category (orange – shaded stimuli).

Figure S14: Multidimensional scaling for the high – gamma neural dissimilarity matrices

A.

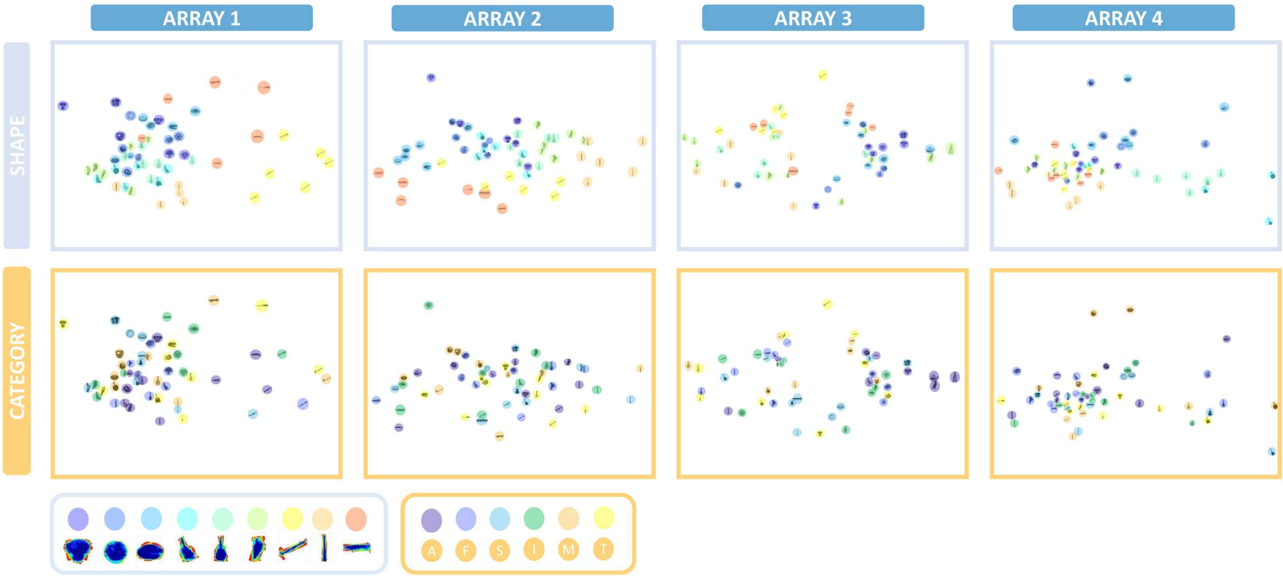

B.

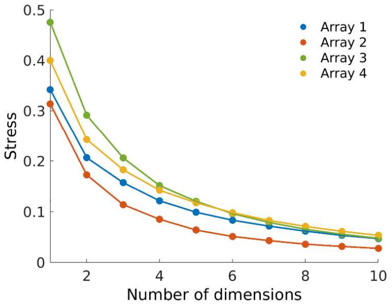

Figure S14: A) MDS performed on the high - gamma neural dissimilarity matrices shows pairwise distances in a 2D space for each array. The 2D arrangements are color-coded first according to the 9 different shape – types (upper panel), and then according to the 6 different semantic categories (lower panel). **B)** Stress level / goodness of fit of the MDS at the MUA level for 1 to 10 dimensions.

Figure S15: Linear decoding of the high – gamma responses

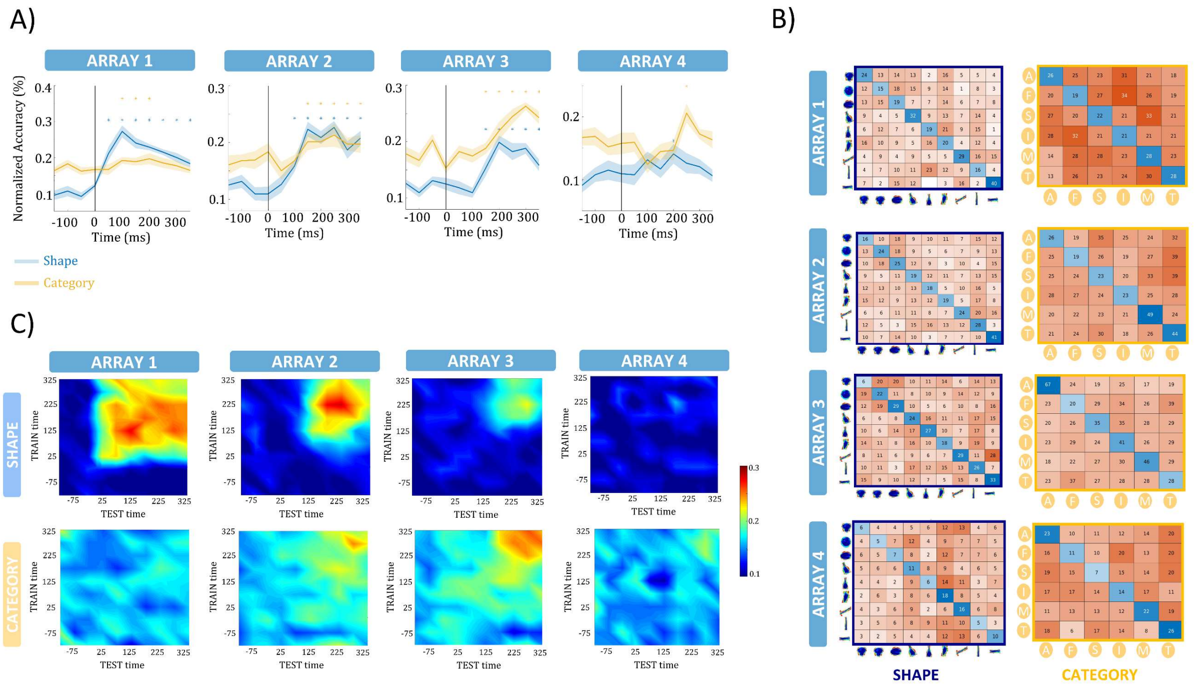

Figure S15: A) Temporal evolution of the SVM decoding accuracy for the shape (blue) and the category (orange) dimension at the high – gamma level. The shaded region around the line represents the standard error across the cross validations. Asterisks (*) indicate significance of decoding accuracy. **B)** Confusion matrices illustrate the performance of decoding per class for the shape (left column) and the category (right column) dimension for a specific time – window (arrays 1,2: 75 -275 ms, array 3: 175 – 275 ms, array 4: 125 – 225 ms) at the high - gamma level. The classification performance of array 3 for the category dimension is predominantly restricted to the "animals" category. **C)** Generalization of the decoders over time for the shape (upper panel) and the category (lower panel) dimension. The y – axis corresponds to the TRAIN time window, the x – axis to the TEST time – window and the colors to the accuracy level of the decoding.

Figure S16: Linear decoding - Control for shape - category information contamination

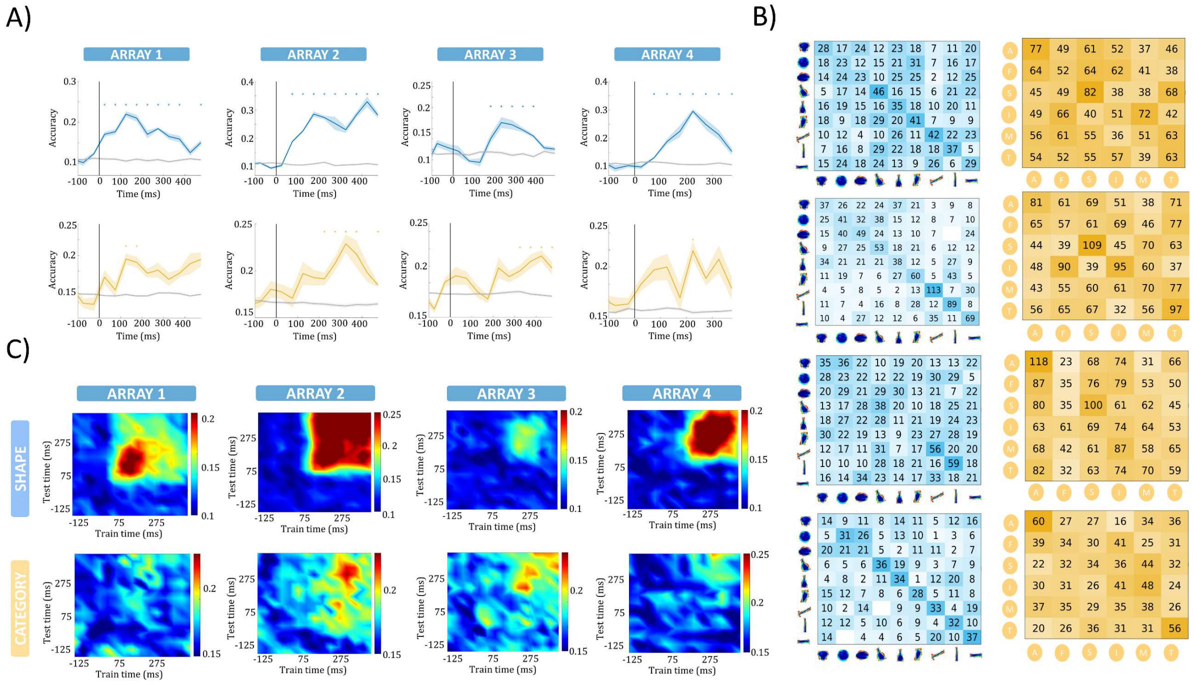

Figure S16: A) Temporal evolution of the SVM decoding accuracy for the shape (blue) and the category (orange) dimension at the MUA level after controlling for shape contamination in category decoding and vice versa. The shaded region around the line represents the standard error across the cross validations. The asterisks (*) indicate significance of decoding accuracy. **B)** Confusion matrices are illustrating the performance of the decoding per class for the shape (left column) and the category (right column) dimension for a specific time – window (arrays 1,2: 75 -275 ms, array 3: 175 – 275 ms, array 4: 125 – 225 ms) at the high - gamma level. **C)** Generalization of the decoders over time for the shape (upper panel) and the category (lower panel) dimension. The y – axis corresponds to the TRAIN time window, the x – axis to the TEST time – window and the colors to the accuracy level of decoding.

Figure S17: Decoding accuracy after removing the “Animals” class

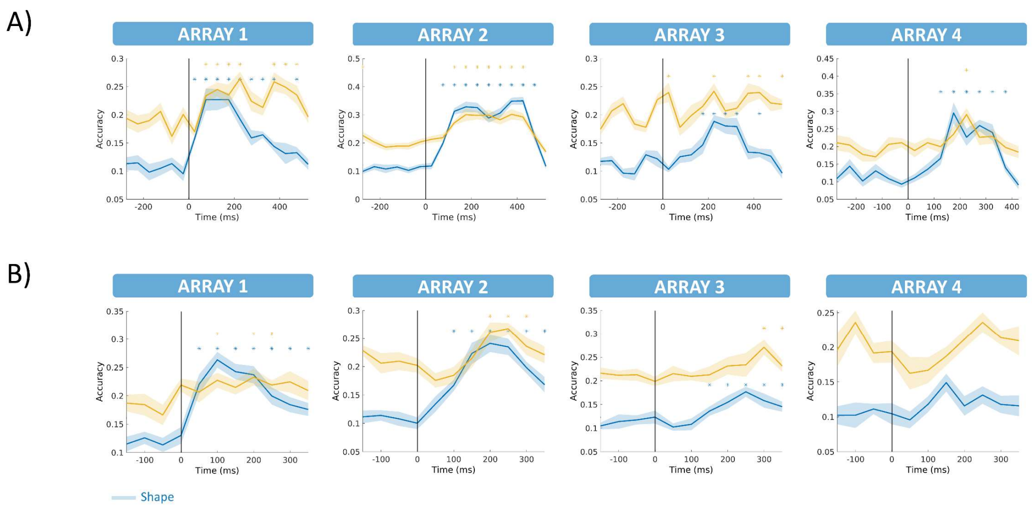

Figure S17: Temporal evolution of the SVM decoding accuracy for the shape (blue) and category (orange) dimension at the MUA (A) and high – gamma (B) level after removing the “Animals” class. The shaded region around the line represents the standard error across the cross validations. The asterisks (*) indicate significance of decoding accuracy. For arrays 3 and 4 the accuracy is considerably lower and less significant without the “Animals” category.

Figure S18: Post – operative scans

A.

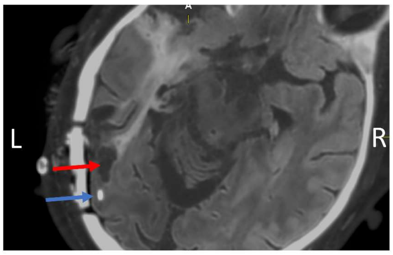

B.

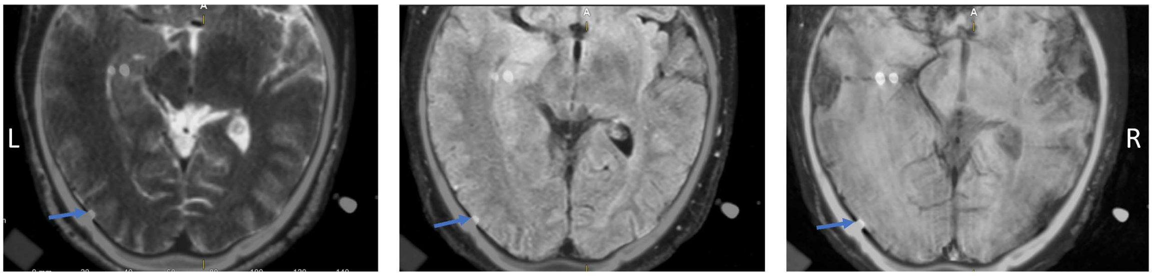

Figure S18: A) Patient 1: Location of the Utah array (blue arrow, hyperdense structure), posteriorly to a previous venous infarction (red arrow), based on a fusion of the CT (during implantation) with the MRI FLAIR images (after removal of the electrodes) (the position of the Utah array does not perfectly match the cortical surface of the MRI due to brain shift of the intraoperative CT). Note that no FLAIR abnormalities are seen in the area. **B)** Patient 2: Location of the Utah array (blue arrow, hyperdense structure), based on the fusion of the postoperative CT (during implantation) with the MRI (after removal of electrodes) T2WI (left), FLAIR (middle) and SWI images (right). No radiographical abnormalities were seen. Abbreviations: L, left; R, right.

Table S1: Summary of the 2 – way ANOVA statistics for the example sites in Figure 2

Example site	SS	df	MS	F	eta^2	p – value
MUA_1 - Shape	19237	8	2404.6	6.34	0.05	0.000
MUA_1 - Category	1978	5	395.6	1.04	0.005	0.52
MUA_1 - Interaction	16827	40	420.7	1.11	0.04	0.65
MUA_2 - Shape	3421	8	427.7	1.86	0.014	0.06
MUA_2 - Category	2832	5	566.5	2.46	0.011	0.02
MUA_2 - Interaction	17036	40	425.9	1.85	0.069	0.0007
MUA_3 - Shape	84939	8	10617	4.43	0.03	0.00003
MUA_3 - Category	11171	5	2234	0.93	0.004	0.46
MUA_3 - Interaction	322515	40	8062	3.36	012	0.000
LFP_1 - Shape	1215167	8	151895.9	6.07	0.06	0.000
LFP_1 - Category	191338	5	38267.7	1.53	0.009	0.17
LFP_1 - Interaction	1280152	40	32003.8	1.28	0.06	0.11
LFP_2 - Shape	1577496	8	197187.1	8.8	0.05	0.16
LFP_2 - Category	2310706	5	462141.3	20.6	0.07	0.000
LFP_2 - Interaction	5853641	40	146341	6.53	0.18	0.000
LFP_3 - Shape	771272	8	96409	4.06	0.04	0.0001
LFP_3 - Category	241338	5	48267	2.03	0.01	0.07
LFP_3 - Interaction	1675346	40	41883	1.76	0.08	0.003

Table S2: Slopes and Confidence Intervals of the ranking analysis

	SLOPE MUA	95 % CI MUA	SLOPE HG	95 % CI HG	SLOPE LFP	95 % CI LFP
**ARRAY 1 - CATEGORY**	-0.22	[-0.32,-0.11]	-0.1	[-0.32,-0.10]	-0.03	[-0.22,0.35]
**ARRAY 1 - SHAPE**	-0.12	[-0.17,-0.05]	-0.1	[-0.17 -0.03]	-0.04	[-0.05 0.13]
**ARRAY 2 - CATEGORY**	-0.18	[-0.23, -0.12]	-0.14	[-0.35, 0.06]	-0.0004	[-0.33,0.33]
**ARRAY 2 - SHAPE**	-0.1	[-0.13, 0-0.07]	-0.09	[-0.15, -0.02]	-0.004	[-0.11 0.10]
**ARRAY 3 - CATEGORY**	-0.17	[-0.28,-0.07]	-0.15	[-0.33,0.03]	0.02	[-0.27, 0.32]
**ARRAY3 - SHAPE**	-0.12	[-0.14,-0.09]	-0.07	[-0.14,0.00]	-0.01	[-0.12, 0.09]
**ARRAY 4 - CATEGORY**	-0.18	[-0.26, -0.10]	-0.1	[-0.37, 0.18]	0.002	[-0.27, 0.27]
**ARRAY 4 - SHAPE**	-0.1	[-0.13, -0.06]	-0.01	[-0.12, 0.09]	0.06	[-0.04, 0.16]

Table S3: Results of Representational Similarity Analysis (RSA) conducted on the high-gamma neural dissimilarity matrices. Spearman's correlation and corresponding p – values following a permutation test (n = 1000).

ARRAYS	Category	Shape	Silhouette
1	Rho = -0.01, p = 0.66	Rho = 0.02, p = 0.21	Rho = 0.13, p = 0.00
2	Rho = -0.01, p = 0.6	Rho = 0.14, p = 0.00	Rho = 0.01, p = 0.36
3	Rho = 0.03, p = 0.15	Rho = 0.27, p = 0.00	Rho = 0.19, p = 0.00
4	Rho = 0.01, p = 0.34	Rho = 0.06, p = 0.006	Rho = 0.14, p = 0.00

Table S4: Maximum correlation between neural RDMs and VGG – 19 RDMs and corresponding layer

**VGG – 19**	LAYER	CORRELATION
ARRAY 1 - MUA	15	0.35
ARRAY 2 - MUA	15	0.32
ARRAY 3 - MUA	15	0.2
ARRAY 4 - MUA	15	0.07
ARRAY 1 - LFP	15	0.30
ARRAY 2 - LFP	14	0.32
ARRAY 3 - LFP	17	0.27
ARRAY 4 - LFP	1	0.10

Table S5: Maximum correlation between neural RDMs and VGG – 19 RDMs and corresponding layer

**RESNET50**	LAYER	CORRELATION
ARRAY 1 - MUA	44	0.47
ARRAY 2 - MUA	42	0.42
ARRAY 3 - MUA	45	0.23
ARRAY 4 - MUA	4	0.19
ARRAY 1 - LFP	44	0.42
ARRAY 2 - LFP	44	0.38
ARRAY 3 - LFP	42	0.28
ARRAY 4 - LFP	4	0.17
